# R-Ras Regulates Murine T Cell Migration and Intercellular Adhesion Molecule-1 Binding

**DOI:** 10.1371/journal.pone.0145218

**Published:** 2015-12-28

**Authors:** Xiaocai Yan, Mingfei Yan, Yihe Guo, Gobind Singh, Yuhong Chen, Mei Yu, Demin Wang, Cheryl A. Hillery, Andrew M. Chan

**Affiliations:** 1 Department of Pediatrics, The Medical College of Wisconsin, Milwaukee, Wisconsin, United States of America; 2 School of Biomedical Sciences, The Chinese University of Hong Kong, Shatin, Hong Kong SAR; 3 Blood Research Institute, BloodCenter of Wisconsin, Milwaukee, Wisconsin, United States of America; 4 Department of Oncological Sciences, The Mount Sinai School of Medicine, New York, New York, United States of America; Candiolo Cancer Institute, ITALY

## Abstract

The trafficking of T-lymphocytes to peripheral draining lymph nodes is crucial for mounting an adaptive immune response. The role of chemokines in the activation of integrins via Ras-related small GTPases has been well established. R-Ras is a member of the Ras-subfamily of small guanosine-5’-triphosphate-binding proteins and its role in T cell trafficking has been investigated in R-Ras null mice (*Rras* ^**−/−**^). An examination of the lymphoid organs of *Rras* ^**−/−**^ mice revealed a 40% reduction in the cellularity of the peripheral lymph nodes. Morphologically, the high endothelial venules of *Rras* ^**−/−**^ mice were more disorganized and less mature than those of wild-type mice. Furthermore, CD4^+^ and CD8^+^ T cells from *Rras* ^**−/−**^ mice had approximately 42% lower surface expression of L-selectin/CD62L. These aberrant peripheral lymph node phenotypes were associated with proliferative and trafficking defects in *Rras* ^**−/−**^ T cells. Furthermore, R-Ras could be activated by the chemokine, CCL21. Indeed, *Rras* ^**−/−**^ T cells had approximately 14.5% attenuation in binding to intercellular adhesion molecule 1 upon CCL21 stimulation. Finally, in a graft-versus host disease model, recipient mice that were transfused with *Rras* ^**−/−**^ T cells showed a significant reduction in disease severity when compared with mice transplanted with wild-type T cells. These findings implicate a role for R-Ras in T cell trafficking in the high endothelial venules during an effective immune response.

## Introduction

R-Ras is a member of the *RAS* superfamily of small guanosine-5’-triphosphate (GTP)-binding proteins with approximately 56% amino-acid sequence homology to Ras oncogenes. In addition to its transforming activity [1, 2], R-Ras is involved in cell spreading [3, 4], actin cytoskeletal organization [5, 6], and migration [7, 8]. The effects of R-Ras on these adhesion-based events are attributed to its ability to activate integrins. By means of an inside-out signaling mechanism, R-Ras simulates the affinity of multiple integrins, including α_5_β_1_ [9], α_IIb_β_3_ [10], and α_M_β_2_ [11]. One plausible mechanism is related to the localization of R-Ras to the endoplasmic reticulum [12]. R-Ras stimulates calcium release at the endoplasmic reticulum, which in turn activates the calcium-binding protease calpain and leads to the cleavage of talin and the activation of integrin [13]. In addition, R-Ras is also enriched in both early and recycling endosomes and promotes exocytosis via the RalA GTPase [14].

Classical Ras proteins are activated by growth factors stimulated receptor tyrosine kinases. In contrast, the upstream regulators of R-Ras belong to a class of receptor that has been implicated in chemoattraction and chemorepulsion. During the process of axon guidance, the chemorepellant Semaphorin 4D binds and activates its receptor, Plexin-B1, which possesses a GTPase-activating protein (GAP) domain that binds R-Ras [15]. The conversion of R-Ras from a GTP- to guanosine diphosphate (GDP)-bound state inactivates this G-protein in a ligand-dependent manner. The subsequent down-modulation of β1 integrin leads to growth cone collapse and axon retraction in the developing nervous system. Independently, the cell-cell adhesion modulator, Notch1, activates R-Ras and stimulates cell adhesion via β1 integrin [16]. Thus, R-Ras appears to be conveying signals from cell-cell contacts to integrin activation.

The physiologic functions of R-Ras have not been fully elucidated. R-Ras knockout mice develop normally without gross morphologic aberrations [17, 18]; however, they display greater angiogenic responses after arterial injury or tumor induction [17]. These vascular phenotypes are attributed to the ability of R-Ras to suppress the proliferation and motility of vascular smooth muscle cells and endothelial cells. It has also been proposed that R-Ras positively regulates the maturation and functional integrity of endothelial cells [19]. Independently, R-Ras knockout mice have defects in Rac-mediated migration and homing of hematopoietic progenitors [20]. Furthermore, R-Ras-null dendritic cells (DCs) form compromised immunologic synapses with antigen-specific T cells [18]. However, the relevance of R-Ras in other immune cell types remains to be explored.

Peripheral lymph nodes (PLNs) are key conduits for mounting an immune response. T cell trafficking in the high endothelial venules (HEVs) is a highly choreographed process that involves dynamic interactions between T cells and endothelial cells [reviewed in [21]]. Four well-defined stages have been delineated, including tethering, rolling, firm adhesion, and diapedesis. Mechanistically, the signaling events that control firm adhesion have been studied extensively. These involve the activation of the chemokine receptors CXCR4 and CCR7 on T cells by CXCL12 and CCL21, respectively [reviewed in [22]]. Via an inside-out signaling mechanism, receptor activation enhances the affinity and avidity of both lymphocyte function-associated (LFA)-1/α_L_β_2_ and very late antigen (VLA)-4/α_4_β_1_ integrins toward intercellular adhesion molecule (ICAM)-1 and vascular cell adhesion molecule (VCAM), respectively. More recent evidence has suggested a role of DCs in promoting the maturation of HEVs via the production of lymphotoxins [23]. Mice with depleted of DCs have reduced cellularity, immature HEVs, and attenuated lymphocyte homing to PLNs [23]. This study reports a novel role for R-Ras GTPase in T cell trafficking and activation. We found that R-Ras knockout mice have smaller lymph nodes and immature HEVs. These phenotypes are correlated with functional defects in proliferation, migration, and activation of R-Ras knockout T cells.

## Materials and Methods

### Mice

The generation of the R-Ras knockout mouse strain, *Rras* ^tm1amc^ (*Rras* ^**−/−**^) was described [18]. Balb/cJ mice were obtained from the Jackson Laboratory (Bar Harbor, ME). The experimental mice used were 5 to 8 weeks of age and were maintained and bred under specific pathogen-free conditions. Female mice were used for most of the experiments described in this study. Euthanasia was carried out by carbon dioxide inhalation at 1L/min until unconsciousness, increased to 2L/min until the cessation of breathing for more than 30 s. This was followed by cervical dislocation to ensure death.

### Ethics Statement

All animal procedures were approved by the Institutional Animal Care and Use Committee of the Mount Sinai School of Medicine (97–594), the Institutional Animal Care and Use Committee of the Medical College of Wisconsin (AUA1328, AUA1460, AUA1782), and the Animal Experimentation Ethics Committee of the Chinese University of Hong Kong (AEEC 15-001-GRF). Humane endpoints included weight loss of more than 20%, lethargy for 3 days, and clinical signs such as uncontrolled diarrhea, alopecia, and inflammation in both eyes that did not resolve. The level of weight loss for a typical graft-versus-host disease (GVHD) experiment is approximately 20% and can vary from experiment to experiment. In our study, our strain of mice followed the classical course of GVDH with an initial loss of body weight followed by a period of recovery. However, in our mouse strain, the weight loss was approximately 25%. The other humane endpoints were not exceeded. Health monitoring was carried out once per day. To minimize suffering and distress, food was placed inside the cages for easy access. Additional soft bedding materials were also added. No pain relievers or anesthesia were administered. No animals died without euthanasia and humane endpoints were used before to the end of the survival experiments.

### Lymph Organ Cellularity and Flow Cytometry

To determine the cellularity of lymphoid organs and the expression of surface markers, the thymus, spleen and PLNs from wild-type or *Rras* ^**−/−**^ mice were collected and homogenized on a 60-mesh stainless steel screen to generate single cell suspensions. Red blood cells were lysed with ACK lysis buffer (Life Tech). The total cell number of each organ was counted using the trypan blue exclusion method on a hemacytometer. The cells were then stained with the appropriate surface markers and analyzed on a Calibur flow cytometer (BD). The data were analyzed with the Flowjo software. All antibodies were purchased from eBioscience.

### Graft Versus Host Disease Induction

Balb/c mice were given a lethal dose of irradiation at 900 rad (9 Gy), and these animals were transfused without or with splenocytes that were adjusted to include 1 × 10^6^ T cells from either wild-type or *Rras* ^**−/−**^ C57BL/6 animals on day 2. Simultaneously, the host immune system was restored by transfusion of 5 × 10^6^ bone marrow cells from wild-type C57BL/6 mice. This allogeneic bone marrow transfusion was critical to avoid the rejection of the infused T cells isolated from C57BL/6 mouse strains. Survival and weight change were monitored daily. At day 21 after transplantation, intestinal tissues were fixed in formalin and embedded in paraffin. Four-micrometer sections were prepared and stained with hematoxylin and eosin.

### MLR Assay

T cell-depleted splenocytes (APCs) from Balb/c mice were isolated, irradiated (3000 cGy), and used as stimulating cells. T-cells (2 × 10^5^) from the spleens of *Rras* ^**+/+**^ or *Rras* ^**−/−**^ mice (129Sv) were isolated by mouse T cell enrichment columns (R&D Systems) and mixed in triplicates with the APCs in different ratios for 4 d. Proliferation was monitored by adding [H^3^]-labeled thymidine (1 μCi per well in a 96-well plate) for the last 16 h. The cells were harvested, and the amount of radioactive materials incorporated was counted in triplicate with a scintillation counter.

### *In Vivo* T Cell Migration Assay

Total T cells were purified with magnetic AutoMACS after incubation with anti-CD4 and -CD8 microbeads (Miltenyi). PKH26 and carboxyfluorescein succinimidyl ester (CFSE) were used to label T cells from wild-type or *Rras* ^**−/−**^ animals, respectively. For PKH26 labeling, 5 × 10^6^ cells/ml in an iso-osmotic solution supplied by the manufacturer (Sigma) were incubated with 3 μM PKH26 at room temperature for 5 to 10 min. Staining was quenched by adding an equal volume of fetal bovine serum (FBS). The cells were then washed once with serum-free RPMI medium. For CFSE labeling, 5 × 10^6^/ml T cells were incubated in RPMI containing 0.5 μM CFSE for 15 min at 37°C. Staining was quenched by an equal volume of 10% FBS in RPMI. The cells were then washed twice with serum free RPMI. An equal number of T cells from each group were mixed together, and a total of 10 × 10^6^ cells was given to wild-type mice by intravenous injection. The mice were euthanized 2 h later; cell suspensions from peripheral blood, spleen, and lymph nodes were prepared; and the percentage and absolute number of infused cells were tracked with flow cytometry.

### *In Vivo* Proliferation of Splenocytes

Naïve C57BL/6 mice were sublethally conditioned with 600 rad of irradiation and received an intravenous infusion of 8 to 10 × 10^6^ CFSE-labeled total splenocytes from wild-type or *Rras* ^**−/−**^ syngeneic donors through the tail vein. Secondary lymphoid organs, including the spleen and inguinal lymph nodes, were collected at specified time points after infusion. The proliferation and migration of infused cells were monitored by dye dilution and quantified based on the percentage of infused cells and the total organ cell number.

### LFA-1 Expression on T Cells

All antibody reagents were purchased from eBiosciences. Total splenocytes were isolated from 7- to 8-week-old-mice. Staining was carried out with allophycocyanin (APC)-CD4 (clone GK1.5), APC-CD8a (clone 53.6.7), phycoerythrin (PE)-CD18 (clone M18/2), and PE-CD11a (Clone M17/4). Six mice were used for each group. Flow cytometry analysis was performed and the mean fluorescence intensities (MFI) of both subunits of LFA-1 in the CD4 and CD8 cell populations were quantified.

### Immunohistochemistry

To reveal the HEV, immunohistochemical analysis was conducted on axillary and inguinal lymph nodes derived from wild-type or *Rras* ^−/−^ mice that were fixed in 10% formalin and embedded in paraffin. Sections with a thickness of 5-μm were subjected to antigen retrieval procedures by incubation in 10 mM citric acid buffer for 20 min followed by 3% hydrogen peroxide for 20 min. The tissue sections were blocked by phosphate-buffered saline solution (PBS) with 10% bovine serum albumin (BSA) for 30 min and then incubated with biotin-conjugated anti-PNAd antibody (1:50 dilution; Biolegend) at 4°C overnight. The sections were then incubated with horseradish peroxidase (HRP)-conjugated streptavidin, and a DAB substrate kit (Vector Lab) was used for color development. Counterstaining was omitted for better viewing of the glycan staining. Bright field images were taken using both 5× and 20× objectives on an Axiovert 200 M microscope (Zeiss). The staining intensity was quantified on a predetermined intensity scale of 1 to 4. Five lymph nodes for each genotype (approximately 15 HEV per node) were analyzed. The data are presented as the mean staining intensity.

### R-Ras Activation by Chemokines

Jurkat T cells were obtained from American Type Tissue Culture Collection (ATCC). Total T cells were purified through magnetic AutoMACS (Miltenyi) after incubation with anti-CD4 and anti-CD8 microbeads. The T cells were triggered with 0.5 μg/ml CCL21 for the indicated time period and solubilized in lysis buffer containing 50 mM Tris-HCl (pH 7.5), 1% NP-40, 200 mM NaCl, 5 mM MgCl_2_, and 5% glycerol. Approximately 0.5 mg of lysates per reaction was incubated with 25 μl glutathione-Sepharose 4B beads (GE Healthcare) coupled with approximately 40 μg of GST-RalGDS-RBD fusion protein and incubated for 1 h at 4°C [18]. Reactions were washed three times with lysis buffer, and the beads were boiled in 60 μl of 2× Laemmli buffer.

### ICAM-1 Binding Assays

*In vitro* soluble ICAM-1 binding analysis was performed as described [24]. Briefly, total T cells were purified by AutoMACS from splenocytes of either wild-type or *Rras* ^**−/−**^ mice. The cells were washed and resuspended in cation-free H/H medium (Hank’s balanced salt solution [HBSS] including 2.0 mg/ml BSA and 10 mM HEPES, pH 7.4). T cells were then stimulated without or with either 0.5 μg/ml of CCL21 for 5 min at room temperature. After washing with the same cation-free medium, the cells were resuspended in binding solution (cation-free medium supplemented with 1.0 mM CaCl_2_ and 1.0 mM MgCl_2_) and incubated with 10 μg/ml of recombinant mouse ICAM-1/Fc or 10 μg/ml Fc fragment of human IgG (R&D Systems) for 30 min at room temperature. A PE-conjugated goat anti-human IgG (eBioscience) was added to the T cells after washing twice with binding solution and incubated at room temperature for another 20 min. The cells were washed three times with PBS and ICAM-1 bound cells were detected with flow cytometry.

For binding to immobilized ICAM-1, each well of a 96-well plate (Costar 3369) was coated with 25 μl of either Fc or ICAM-1/Fc (10 μg/ml) overnight at 4°C. Wells were blocked with PBS and 0.5% BSA for 4 h. Approximately 2 × 10^6^ T cells were seeded per well in triplicate and allowed to incubate for 20 min at 37°C in cation-supplemented binding buffer. Wells were then exposed to either blank buffer or CCL21 (1 μg/ml) and incubated for an additional 20 min. Wells were washed three times with binding buffer. Adherent cells were scrapped and counted with a hemocytometer.

### Cell Adhesion Assay

The SV40-transformed murine endothelial cell line, 2H-11, was obtained from ATCC and maintained in DMEM with 10% FBS. The cells were seeded in 12-well plates and allowed to reach confluence. The cells were placed in low serum (1% FBS) for 6 hours and treated with or without transforming growth factor-alpha (10 ng/ml, TNF-α; R&D Systems) for 4 h. The cells were then rinsed three times with HBSS. Around 5 × 10^5^ CFSE-labeled T cells in 0.5 ml of HBSS were added to each well and incubated for 30 min at 37°C. Unbound cells were removed by washing three times with HBSS. Bound T cells were imaged from three randomly selected fields per well using a 10× objective of an inverted fluorescence microscope (Eclipse Ti-E, Nikon). The number of adherent cells in each field was quantified by ImageJ software.

### Statistical Analysis

Statistical significance was determined using a two-tailed student’s *t* test. The results are expressed as mean values with error bars indicated. The GVHD survival data were analyzed by two-way analysis of variance and the log-rank test. Statistical significance was considered to be *p* values of less than 0.05.

## Results

### Peripheral Lymph Node Phenotypes of *Rras* ^-/-^ Mice

**Fig 1:**
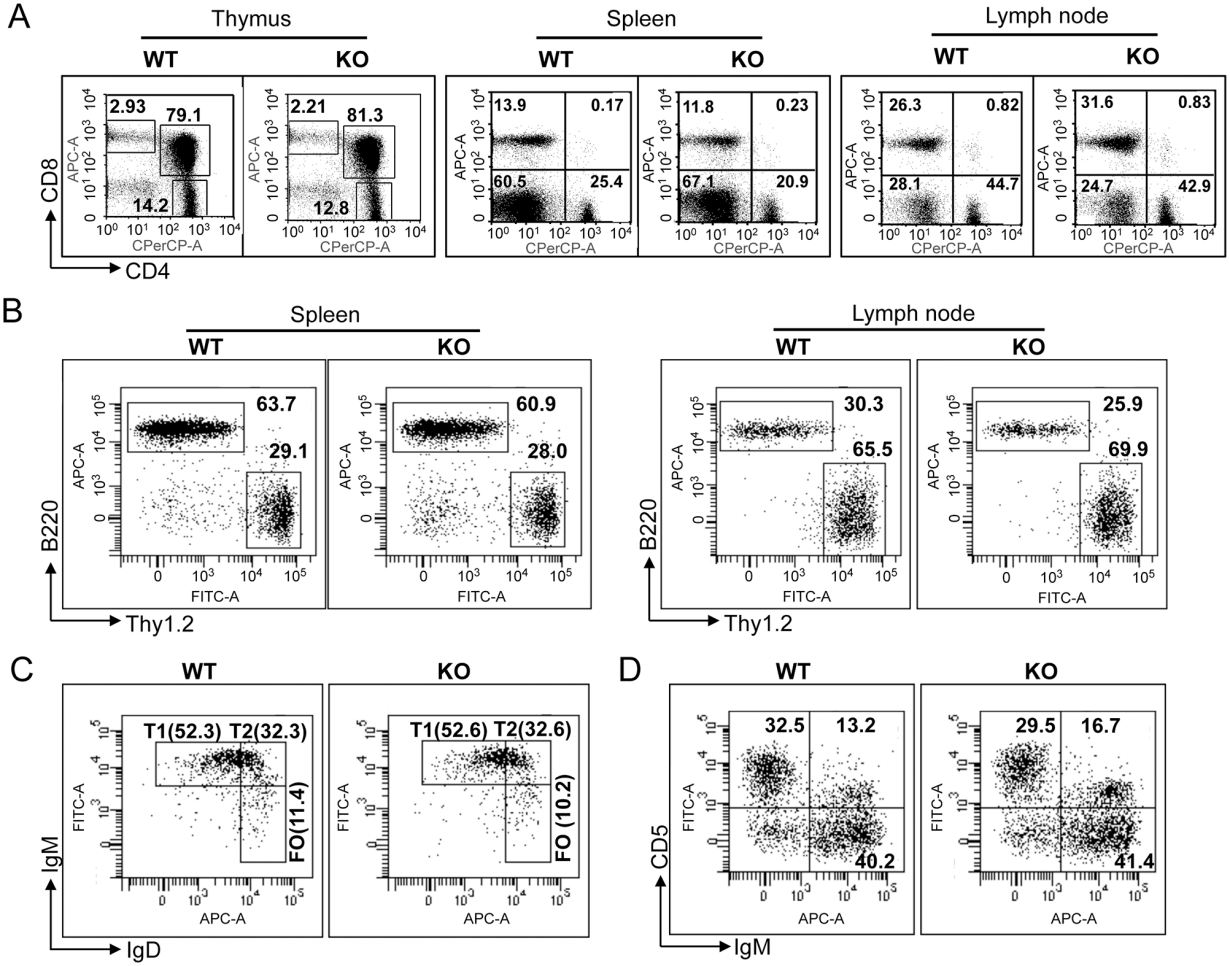
Immune cells development and functions of *Rras* ^−/−^ mice. Indicated lymphoid organs from *Rras* ^**+/+**^ (wild-type; WT) and *Rras* ^−/−^ (knockout; KO) mice were analyzed by flow cytometry for subpopulations of (A) T cells, (B) T and B cells, (C) B220^+^ splenic T1, T2, and FO B cells, and (D) B220^+^ splenic B1 B cells.

To elucidate the role of R-Ras in the developing lymphoid organs, thymus, spleen, and PLNs were isolated from 6- to 8-week-old *Rras* ^**+/+**^ and *Rras* ^−/−^ mice. Flow cytometry analysis of CD4^**+**^ and CD8^**+**^ populations did not reveal aberrant T cell development in *Rras* ^−/−^ mice when compared to *Rras* ^**+/+**^ animals (Fig 1A and S1 Fig). Similarly, *Rras* ^−/−^ mice did not show defects in B-cell development in the spleen and lymph node (Fig 1B). Also, the relative fractions of T1, T2, follicular B cells (Fig 1C), and B1 B cells (Fig 1D) were very similar between the *Rras* ^−/−^ and *Rras* ^**+/+**^ mice.

**Fig 2:**
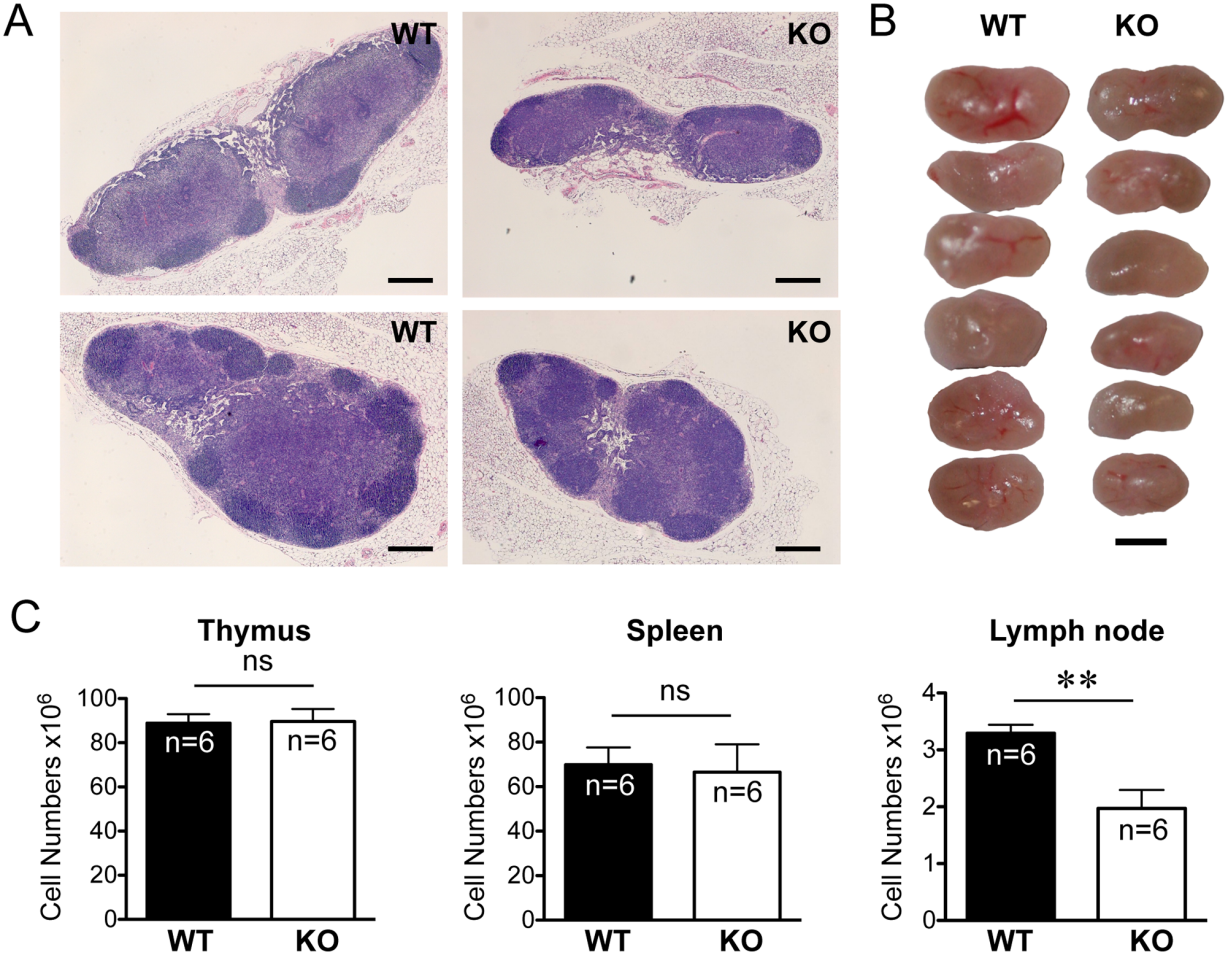
Decreased peripheral lymph organ cellularity in *Rras* ^−/−^ mice. (A) Hematoxylin and eosin staining of axillary lymph nodes. *Bars*, 200 μm. (B) Pictures of lymph nodes are shown. *Bars*, 400 μm. (C) Cell suspensions were prepared from the indicated organs (six in each group) and cellularity was determined by trypan blue exclusion. Data represent at least three independent experiments. Statistical analysis was performed using a two-tailed student’s *t* test. *Bars*, SE. *ns*, no significant difference. **p< 0.005.

Analysis of the cellularity of the lymphoid organs revealed reduced lymph node sizes (Fig 2A and 2B) with an average of a 40% reduction in the total cell numbers (Fig 2C). Morphologically, *Rras* ^−/−^ lymph nodes retained their overall internal architecture, and the follicles in the node cortex were clearly demarcated. However, the T cell regions in the paracortex in which HEV resided were less prominent when compared to those in the wild-type mice (Fig 2A). In contrast, no significant differences in the thymus and spleen size or morphology were seen between the *Rras* ^**+/+**^ and *Rras* ^−/−^ mice (Fig 2C). The *Rras* ^−/−^ mice have body masses similar to those of age-matched wild-type mice (S2 Fig).

### R-Ras Is Required for High Endothelial Venule Development and CD62L Expression in T Cells

**Fig 3:**
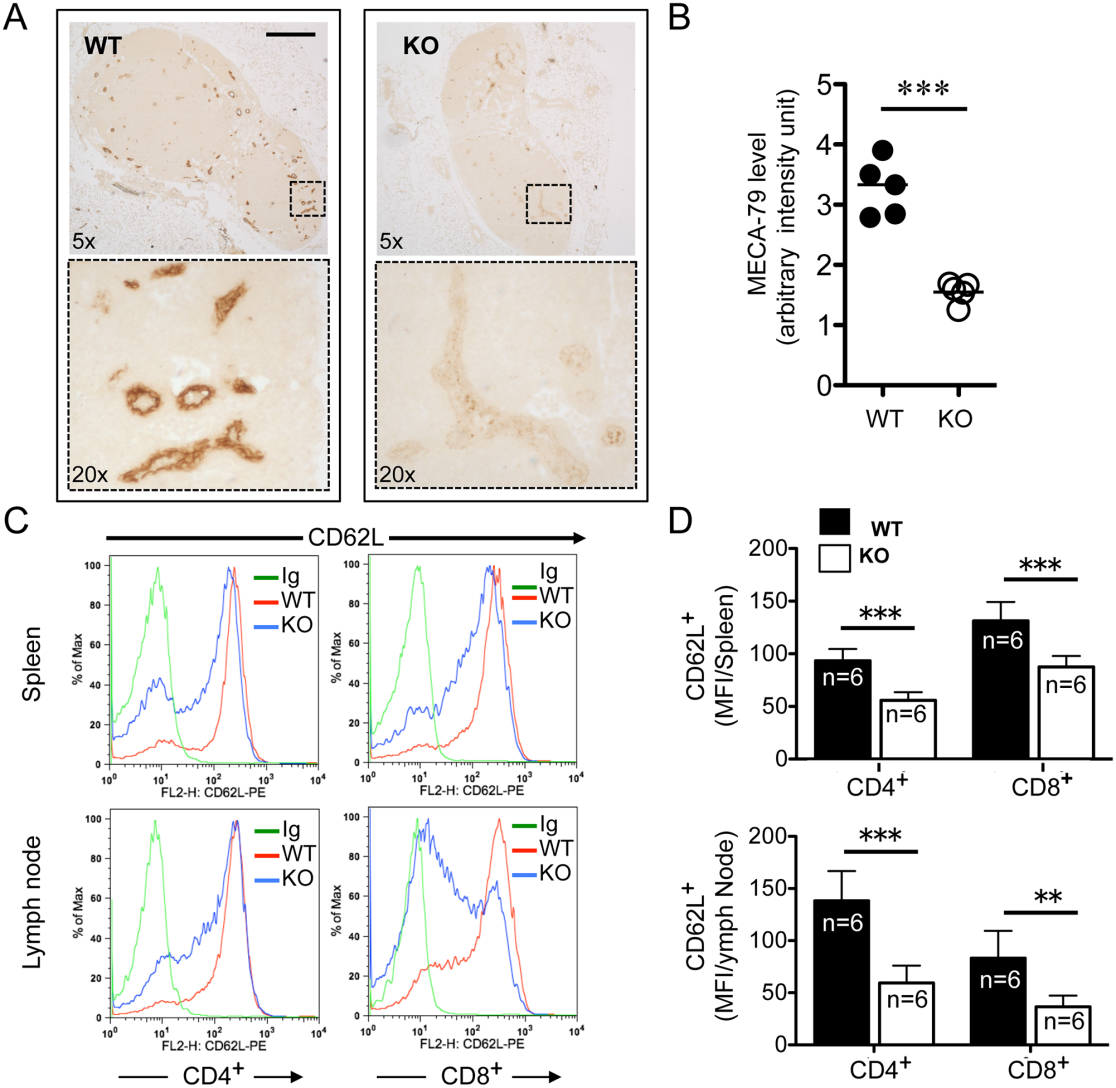
Aberrant lymph nodes in *Rras* ^−/−^ mice. **(**A) Immunohistochemical analysis of *Rras* ^**+/+**^ (WT) and *Rras* ^−/−^ (KO) lymph nodes stained with anti-MECA-79 (*brown*). Magnified areas (*boxed*) are shown in lower panels. *Bars*, 200 μm. (B) Staining intensity was quantified from five lymph nodes. *Bars*, means. ***p<0.0001, *t*-test. (C) Surface expression of CD62L in CD4^**+**^ and CD8^**+**^ T cells from spleen and lymph nodes (LN) of *Rras* ^**+/+**^ and *Rras* ^−/−^ mice was analyzed with flow cytometry. Isotype immunoglobulin (*Ig*) was used as a control. (D) The results from six mice per group were quantified and depicted. *Bars*, SD, two-tailed *t*-test. **p<0.005, ***p<0.0001.

Blocking the maturation of HEVs has been reported to cause atrophic PLNs after depletion of the DC population [23]. For this reason, the expression of a glycoprotein-determinant presented on HEV was analyzed with an anti-MECA-79 antibody, which recognizes a peripheral node-addressin present in mature HEVs. Immunohistochemical (IHC) analysis revealed strong staining of vascular components in the paracortical region of *Rras* ^**+/+**^ PLNs (Fig 3A). For *Rras* ^−/−^ PLNs, although there were similar numbers of HEVs, the intensity of MECA-79 staining was three- to four-fold weaker (Fig 3B). These differences were not likely a result of variations in staining procedures because immunohistochemical analysis was performed on *Rras* ^**+/+**^ and *Rras* ^−/−^ PLN sections immobilized on the same glass slide.

L-selectin (CD62L) is an adhesion molecule on the T cell surface that binds sialylated carbohydrates on HEVs [25]. This interaction is critical for the tethering and rolling of T cells before they enter the paracortical regions of PLNs. To determine whether the reduced cellularity seen in *Rras* ^**-/-**^ PLNs could be due to altered surface expression of CD62L, fluorescence-activated cell sorting (FACS) analysis was performed on T cells isolated from either PLNs or spleens. The expression levels of CD62L in *Rras* ^−/−^ CD4^+^ and CD8^+^ T cells were reduced by 40.8% and 33.6% in the spleen and 57.2% and 56.6% in PLNs, respectively (Fig 3C and 3D). Furthermore, the fractions of CD62L-positive CD4^**+**^ and CD8^**+**^ T populations in *Rras* ^−/−^ mice were reduced by 16.7% and 14.8% in the spleen; and 37.8% and 46.8% in PLNs, respectively (S3 Fig). Thus, defects in HEV maturation and the reduced expression of CD62L in T cells may explain the smaller PLNs observed in *Rras* ^−/−^ mice.

### Impaired T Cell Proliferation in *Rras* ^-/-^ Mice

**Fig 4:**
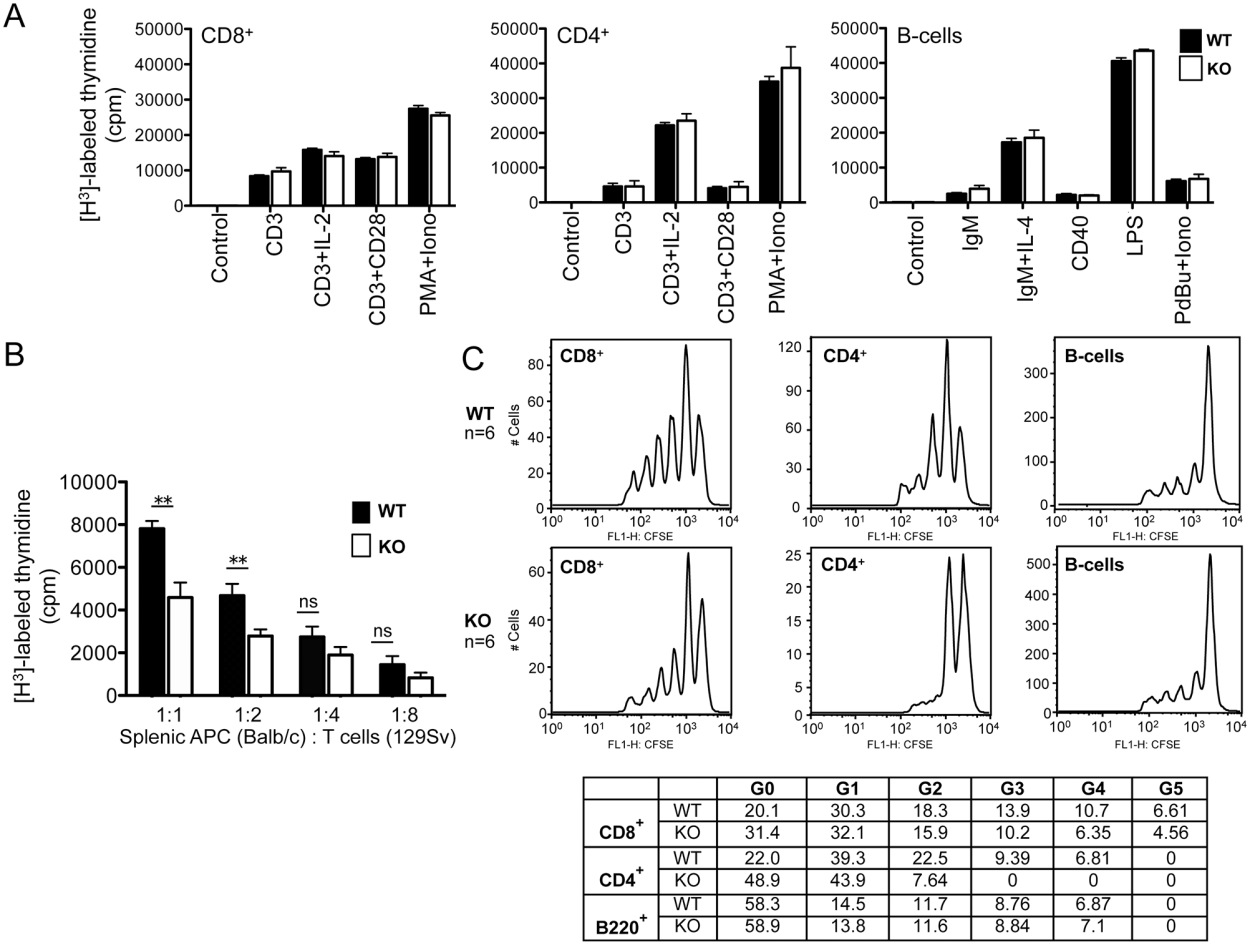
Attenuated T cell proliferation in *Rras* ^−/−^ mice. (A) T and B lymphocytes were stimulated with the indicated immune regulators and cell proliferation was measured by thymidine incorporation techniques. Results from triplicate measurements using two mice per group. *Bars*, SD. Abbreviations, *IL-2*, interleukin-2; *PMA*, phorbol myristate acetate; *Iono*, ionomycin; interleukin-4, IL-4; LPS, lipopolysaccharide; PdBU, phorbol 12, 13-dibutyrate. (B) Mixed lymphocyte reaction assays were carried out by co-mixing T cells from either *Rras* ^**+/+**^ (*solid bars*) or *Rras* ^−/−^ (*open bars*) mice in 129Sv strain with T cell-depleted splenic antigen-presenting cells from WT Balb/c mice at the indicated ratios. T cell proliferation was assessed 4 days later with the [H^**3**^]-thymidine incorporation method. Results represent triplicate measurements from a single experiment that was reproduced twice. *Bars*, SD. *ns*, no significant difference. **p< 0.005. (C) *In vivo* proliferation of splenocytes was conducted by the dye dilution method. Sublethally irradiated naïve C57BL/6 mice were infused with 8 to 10 × 10^6^ of CFSE labeled total splenocytes from either *Rras* ^**+/+**^ (WT) or *Rras* ^−/−^ (KO) mice. After 14 days, spleens were collected for dye dilution assays followed by flow analysis. Representative data are shown with six per group. The relative cell numbers (%) in individual peaks are shown in the table.

The data thus far suggest potential impairment of T cell functions in *Rras* ^−/−^ mice. One possibility for this impairment is the defects associated with T cell activation and proliferation. To test this possibility, the *in vitro* proliferative capacity of CD4^**+**^ and CD8^**+**^ T cells, as well as B-cells, in response to immune modulators was examined. However, no significant differences were observed between *Rras* ^**+/+**^ and *Rras* ^−/−^ mice (Fig 4A). Next, the relative proliferative capacity of *Rras* ^**+/+**^ and *Rras* ^−/−^ T cells co-mixed with allogeneic antigen-presenting cells (APCs) from Balb/c mice were evaluated. There was a 30% to 50% reduction in the proliferation of T cells from *Rras* ^−/−^ mice when compared with wild-type mice over a range of co-mixing ratios (Fig 4B).

To further substantiate our findings, *in vivo* T cell proliferation assays were performed. Sublethally irradiated wild-type mice were infused with 8 × 10^6^ CFSE-labeled total splenocytes. Two weeks later, the extents of dye dilution of the infused T cells in the spleen were analyzed with flow cytometry. Both *Rras* ^−/−^ CD4^**+**^ and CD8^**+**^ T cells have reduced proliferative peaks when compared to *Rras* ^**+/+**^ T cells (Fig 4C). In contrast, both *Rras* ^**+/+**^ and *Rras* ^−/−^ B-cells proliferated to a similar extent. The extent of T cell proliferation was also examined in the PLNs. In contrast to *Rras* ^**+/+**^ splenocytes, transfused T cells from *Rras* ^−/−^ mice were below the threshold of detection in the PLNs of the recipient irradiated *Rras* ^**+/+**^ mice (data not shown). This may reflect the intrinsic homing defects of T cells that lack R-Ras.

### Reduced Homing Capacity of *Rras* ^-/-^ T Cells

**Fig 5:**
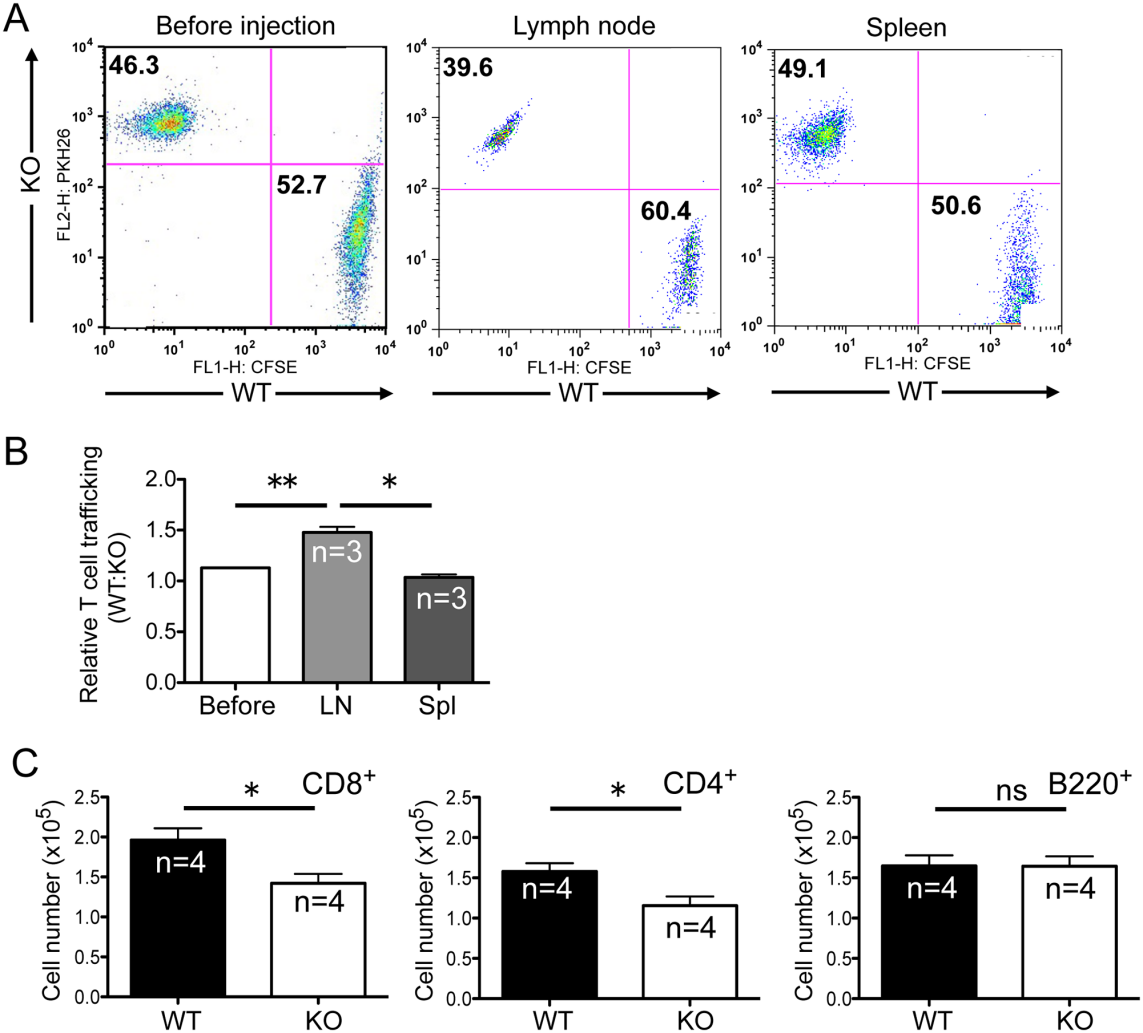
Homing defects of *Rras* ^−/−^ T cells. (A) *Rras* ^**+/+**^ (WT) and *Rras* ^−/−^ (KO) naïve T cells were separately labeled with PKH26 and CFSE, respectively. Then, 5 × 10^6^ cells of each population were co-mixed and injected into WT mice. After 2 h, the uninjected cell mix (*Before*), lymph nodes (*LN*), and spleen (*Spl*) were analyzed with flow cytometry. (B) The relative ratios of *Rras* ^**+/+**^ to *Rras* ^−/−^ T cells are depicted. Results are from a representative experiment and was reproduced twice. *Bars*, SD. Paired *t*-test. *p<0.05, **p<0.005. (C) CFSE-labeled T cells were injected into WT mice, and after 6 days, different lymphocyte subsets in PLNs were quantified by flow cytometry. *Bars*, SE. *p<0.05, ns, not significant.

To clearly demonstrate whether reduced CD62L expression in *Rras* ^−/−^ T cells could affect their homing capacity, *Rras* ^**+/+**^ and *Rras* ^−/−^ T cells were labeled separately with CFSE and PKH26, respectively, and similar numbers were infused together into wild-type syngeneic hosts. The relative amounts of these two T cell populations were quantified after 2 h (Fig 5A). There was a 1.5-fold reduction in the amount of *Rras* ^−/−^ T cells trafficking to the lymph nodes when compared to *Rras* ^**+/+**^ T cell controls (Fig 5B). This difference was not due to aberrant circulation because a similar number of *Rras* ^**+/+**^ and *Rras* ^−/−^ T cells was found in the blood of the recipient animals (not shown). Also, the homing defects of *Rras* ^−/−^ T cells were specific to the lymph node, as all infused T cells displayed a very similar migratory capacity to the spleen (Fig 5B). Separately, total splenocytes were labeled with CFSE and infused into wild-type mice. After 6 d, the relative number of *Rras* ^**+/+**^ and *Rras* ^−/−^ T cell subsets in PLNs was analyzed by flow cytometry. There was a 25% reduction in the number of *Rras* ^−/−^ CD4^**+**^ and CD8^+^ T cells that migrated to the PLNs when compared to *Rras* ^**+/+**^; however, the B220^**+**^ subtype of B-cells was not altered (Fig 5C). These data suggest that defects in HEV maturation and T cell trafficking may contribute to the smaller lymph node size in *Rras* ^−/−^ mice.

### Activation of R-Ras-GTP Loading by CCL21 Chemokine

**Fig 6:**
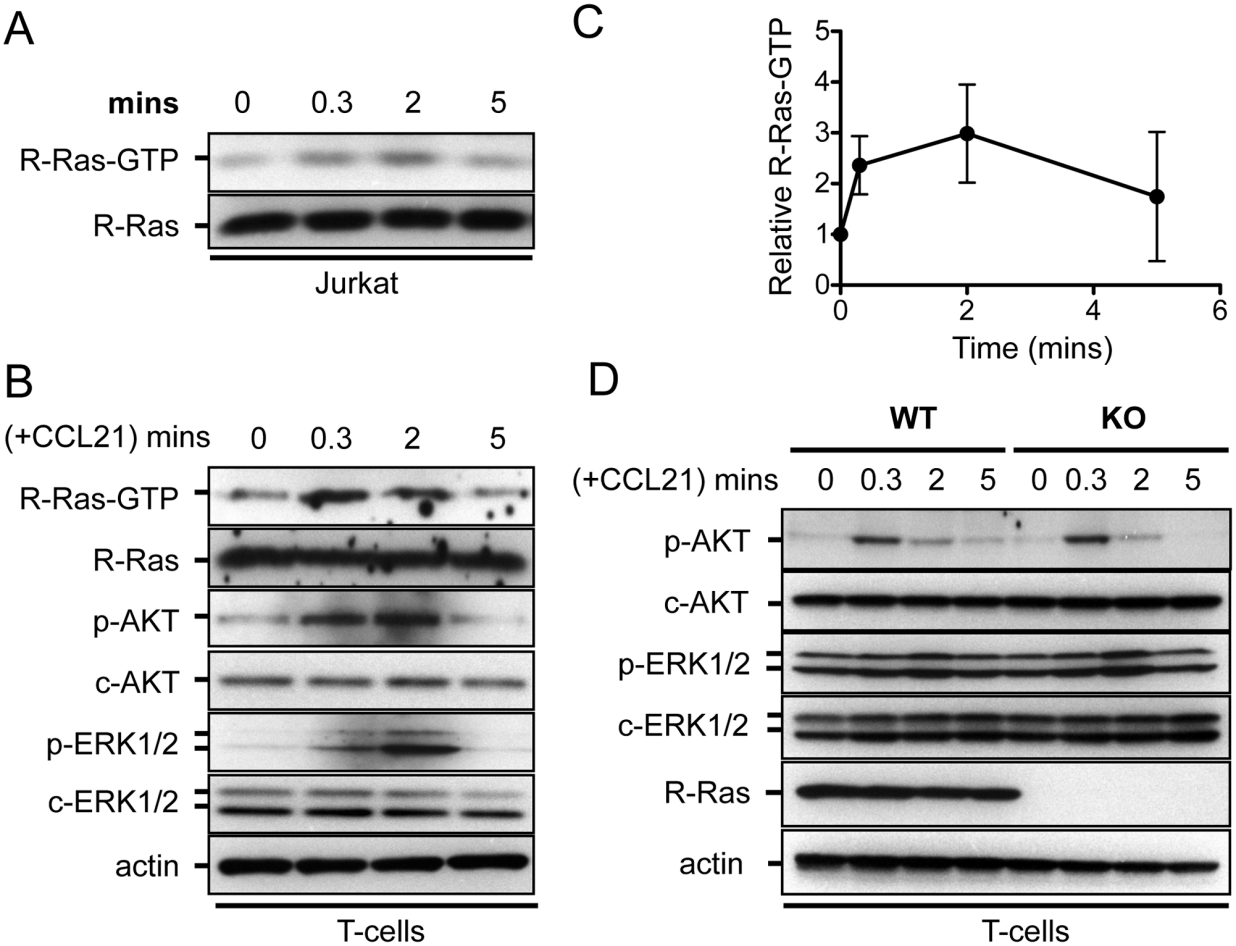
CCL21 activates R-Ras GTP-loading. (A) Jurkat cells and (B) splenic T cells were treated with CCL21 (0.5 μg/ml) for the indicated duration. The levels of GTP-bound active R-Ras were determined by pull-down assays using a RalGDS-RBD affinity probe. The activation states of AKT (*p-AKT*) and ERK1/2 (*p-ERK1/2*) are shown. (C) Results from splenic T cells were quantified from three independent experiments and show the fold-increase in R-Ras-GTP at each time point. *Bars*, SD. (D) T cells from *Rras* ^+/+^ (*WT*) and *Rras* ^−/−^ (*KO*) mice were stimulated with CCL21 (0.5 μg/ml), and the levels of p-AKT and p-ERK1/2 are analyzed with Western blotting analysis. T cells were pooled from two mice per group.

Because chemokines produced by endothelial cells are known to activate integrins in T cells, we tested whether R-Ras could play a role in this process. CCL21 is a chemokine that stimulates the interaction between LFA-1 (α_L_β_2_) integrin and ICAM-1 expressed on endothelial cell surface. To determine whether CCL21 could stimulate the turnover of GTP and GDP of R-Ras, an affinity pull-down assay was performed using Jurkat T cells. CCL21 activated the R-Ras-GTP level by approximately 20 s and peaked around 2 min before returning to a basal level at 5 min (Fig 6A). Next, very similar kinetics of R-Ras activation was also observed in splenic T cells from wild-type animals (Fig 6B and 6C). The rapid kinetics of R-Ras activation closely followed to that of PI3-K, as demonstrated by the level of p-Akt. However, the Erk1/2 pathway was activated later at 2 min (Fig 6B). However, a comparison of the magnitude and kinetics of Akt and Erk1/2 activations in *Rras* ^**+/+**^ T *Rras* ^−/−^ T cells did not revealed substantial differences (Fig 6D).

### Decreased ICAM-1 Binding in *Rras* ^-/-^ T Cells

**Fig 7:**
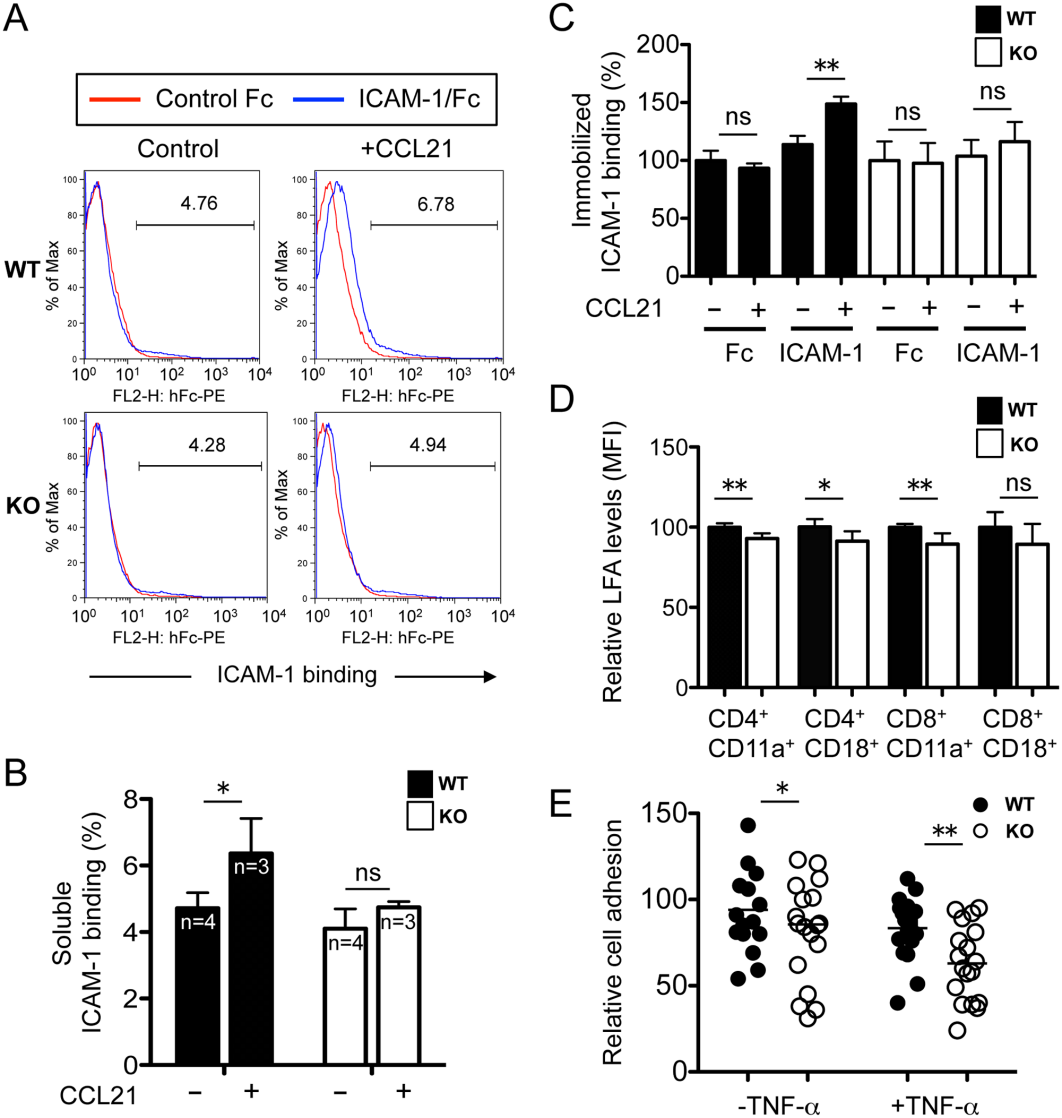
Reduced ICAM-1 binding in *Rras* ^−/−^ T cells. (A) Splenic T cells were treated with CCL21 (0.5 μg/ml) for 5 min, washed and incubated with ICAM-1/Fc (10 μg/ml) for 30 min. Bound Fc was detected by a PE-conjugated goat anti-human IgG followed by flow cytometry analysis. (B) Quantification of results is shown as the percentage of ICAM-1 binding. *Bars*, SD. *p<0.05, two-tailed *t*-test. *ns*, not significant. (C) Quantification of T cells binding to immobilized Fc and ICAM-1/Fc in 96-well plates in the presence or absence of CCL21 (1 μg/ml). Results are presented as ICAM-1 binding relative to untreated controls. Three mice were used per group. *Bars*, SD. Two-tailed *t*-test. **p<0.005, *ns*, not significant. (D) The relative surface expression of CD11a and CD18 on splenic T cells were quantified by FACS. Results are from six animals per group. *Bars*, SD. Two-tailed *t*-test. *p<0.05, **p<0.005, *ns*, not significant. (E) Cell adhesion assays were carried out in 2H-11 endothelial cells. CFSE-labeled T cells were added in triplicates to 2H-11 pretreated without or with TNF-α. Bound cells were quantified by counting the number of green cells from three randomly selected fields. Data are from two mice per group. *Bars*, mean values. Two-tailed *t*-test. *p<0.05, **p<0.005.

Next, the ability of T cells to bind soluble ICAM-1/Fc chimera upon chemokine stimulations was examined. The chemokine CCL21 is known to activate LFA-1 on T cells, which is essential for ICAM-1 binding on endothelial cells of HEV. As expected, CCL21 stimulated soluble ICAM-1-binding in *Rras* ^**+/+**^ T cells by 1.34-fold (Fig 7A and 7B). In contrast, *Rras* ^−/−^ T cells treated with this chemokine were stimulated by 1.16-fold only (Fig 7A and 7B). This represents a 14.3% reduction in soluble ICAM-1 binding in R-Ras-null T cells. In all cases, Fc alone was used as a negative control in these binding studies. We also performed similar binding studies using ICAM-1 immobilized on tissue culture dishes. The addition of CCL21 stimulated binding to ICAM-1 by 1.31-fold for *Rras* ^+/+^ T cells and 1.12-fold for *Rras* ^−/−^ T cells (Fig 7C). Therefore, binding to immobilized ICAM-1 was reduced by 14.5% in R-Ras-null T cells.

The reduced binding of *Rras* ^−/−^ T cells to soluble ICAM-1 may be caused by reduced expression of LFA-1. To test this possibility, the surface expression of the two subunits of LFA-1, CD11a and CD18, was quantified by FACS analysis. As shown in Fig 7D, decreases of 7.0% and 10.5% were seen in the surface expression of CD11a in *Rras* ^−/−^ CD4^+^ and CD8^+^ cells, respectively, and a 8.8% decrease was seen in the CD18 expression in CD4^+^ cells. In contrast, the expression levels of CD18 in CD8^+^ cells were not significantly affected.

To determine whether the reduced binding to soluble ICAM-1 and decreased LFA-1 expression in *Rras* ^−/−^ T cells could perturb adhesion to endothelial cells, adhesion assays were performed using the mouse endothelial cell line, 2H-11 [26]. 2H-11 cells are SV40-transformed endothelial cells derived from the vascular epithelium of axillary lymph nodes. The ability of *Rras* ^−/−^ T cells to adhere to 2H-11 cells was reduced by 21.4% and 24.5% in untreated and TNF-α-treated cultures, respectively (Fig 7E). Unexpectedly, the inflammatory cytokine TNF-α failed to stimulate cell adhesion. It is possible that 2H-11 is insensitive to TNF-α because of its transformed status. Taken together, these findings implicate R-Ras in the regulation of LFA-1 expression and binding to ICAM-1 after chemokine stimulation.

### Reduced Graft-Versus Host Disease in Recipient Mice Induced by *Rras* ^-/-^ T Cells

**Fig 8:**
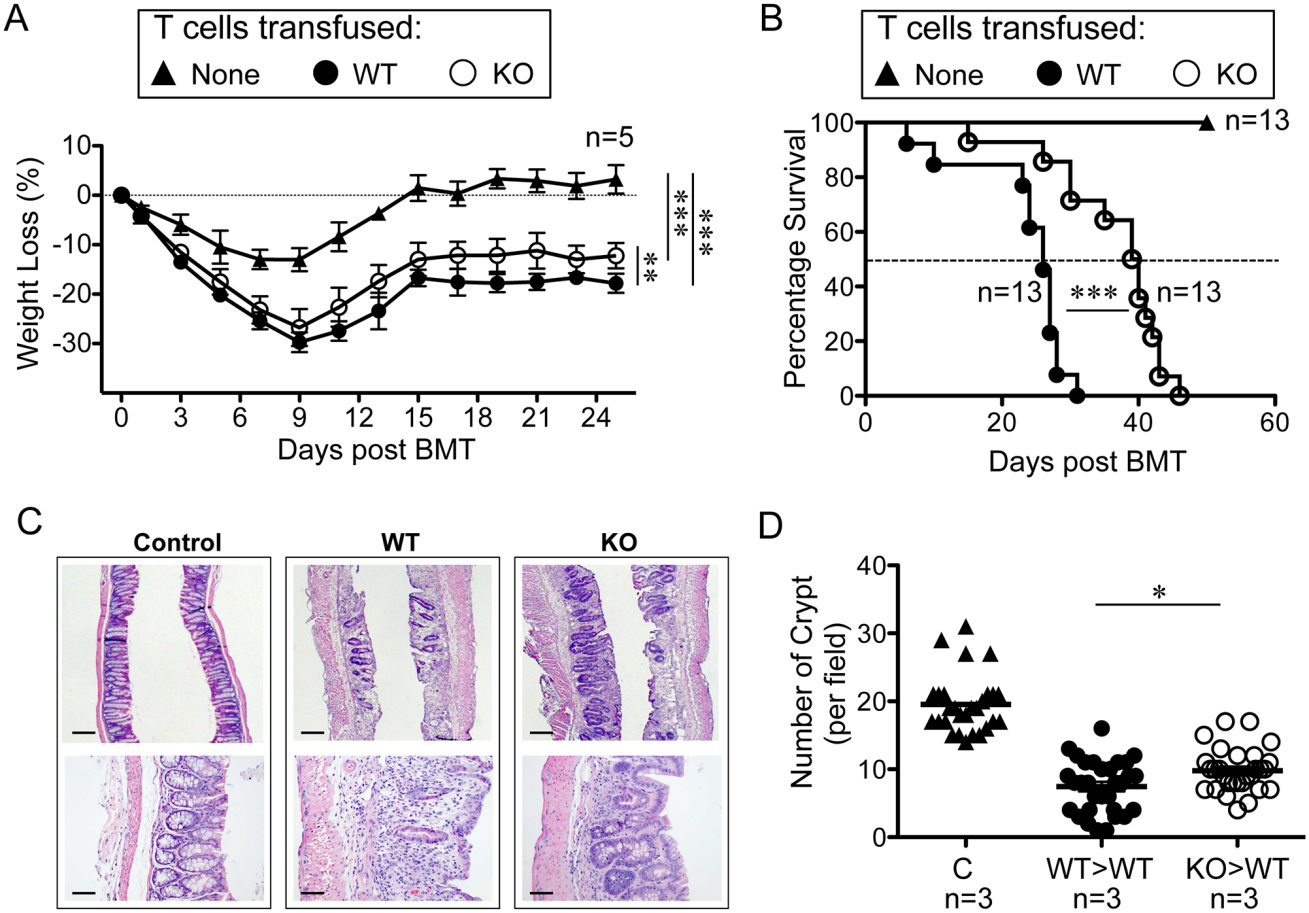
Attenuated Severity of GVHD induced by T cells from *Rras* ^−/−^ animals. Balb/c mice were given 900 rad lethal dose irradiation and received 5 × 10^6^ of bone marrow cells from WT C57BL/6 mice. These animals were also transfused without (▲) or with splenocytes that were adjusted to include 1 × 10^6^ of T cells from either WT (●) or *Rras* ^−/−^ (○) C57BL/6 animals on the second day. Body weight changes (A) and animal survival (B) were monitored. Body weight loss data from a representative experiment is shown (A). *Bars*, SD. One-way ANOVA. **p<0.005, ***p<0.0001. Survival data are presented by combining two independent experiments with 13 mice per group. Statistical analysis was performed using two-way ANOVA (A) and Log-rank (Mantel-Cox) test (B). ***p<0.0001. (C) Hematoxylin and eosin staining of ileal intestines collected on day 21 after T cell transfusion. Upper panels (2.5× *Bars*, 200 μm); lower panels (20× *Bars*, 25 μm). (D) Images were analyzed for the number of crypts per field of view. Approximately 30 fields were analyzed from three mice per group. *Bars*, mean. Unpaired *t*-test. *p< 0.05.

To demonstrate the *in vivo* relevance of R-Ras in T cell functions, GVHD was induced in wild-type Balb/c mice by means of allogeneic transplantation of splenocytes from either *Rras* ^**+/+**^ or *Rras* ^−/−^ mice in the C57BL/6 background. Control mice that did not receive allogeneic T cells fully recovered to their pretransplantation weight within 20 days (Fig 8A). All of the mice that received allogeneic splenocytes developed the characteristic biphasic disease progression seen with GVHD. The mice that received either *Rras* ^**+/+**^ or *Rras* ^−/−^ splenocytes harbored symptoms of GVHD, including diarrhea, cutaneous lesions, and weight loss.

The two experimental groups had very similar initial clinical signs of disease in terms of the extent of weight loss through day 8. Both groups also showed similar clinical improvement from the irradiation from day 8 until day 14 (Fig 8A). However, mice that received *Rras* ^−/−^ T cells lost 5% less weight when compared to mice that received with *Rras* ^**+/+**^ T-lymphocytes. The median survival time for mice that received *Rras* ^−/−^ T cells was 16 d longer when compared to mice received *Rras* ^**+/+**^ T cells (Fig 8B). Histologic analysis of the ileum from recipient mice that received *Rras* ^**+/+**^ T cells revealed extensive inflammation of the epithelium and villi (Fig 8C). Detailed analysis revealed that the relative number of crypts of Lieberkuhn in mice that received either *Rras* ^**+/+**^ or *Rras* ^−/−^ T cells was 7.5±0.7 and 9.7±0.6, respectively (Fig 8D). The extent of damage extended beyond the layers between the mucosa and submucosa for the *Rras* ^**+/+**^ compared to *Rras* ^−/−^. In contrast, the control mice had significantly greater numbers of crypts (19.5±0.8) and retained normal histologic morphology. From these data, we conclude that the GVHD responses induced by allogeneic transplantation of *Rras* ^−/−^ T cells are attenuated in recipient mice.

## Discussions

This study examined the functional role of R-Ras in T cells. We found that R-Ras is not required for T cell development. However, in line with its role in the promotion of integrin activity, R-Ras is required for adhesion-based T cell functions. This requirement is reflected in the reduced lymph node cellularity, defects in T cell homing, and attenuated GVHD observed in *Rras* ^−/−^ mice. In addition, R-Ras-null mice have defects in allogeneic T cell responses.

These results are reminiscent of those in H-ras and N-ras knockout mice; both have normal T cell development but impaired TCR-induced IFN-γ production in CD4^**+**^ T cells [27]. In contrast, TC21, a closely-related GTPase, constitutively interacts with the TCRs and promotes T cell proliferation and survival via the PI3-K pathway [28]. Rap1A, another Ras-related G-protein, is constitutively active in T cells and strongly stimulates both β1 and β2 integrins [29]. Indeed, RapL, a downstream substrate of Rap1A, is required for T cell trafficking to secondary lymphoid organs by mediating adhesion to fibronectin and ICAM-1 [30]. Previous studies have indicated that R-Ras is an upstream regulator of Rap1A in macrophages [11]. In fact, R-Ras has been shown to interact with RapL [30]. Therefore, it is tempting to speculate that R-Ras can regulate T cell trafficking through Rap1A and RapL.

In contrast, the activation of Rap1 is required for T cell anergy by blocking TCR- and CD28-mediated IL-2 gene expression [31]. Whether the attenuation of GVHD observed in *Rras* ^−/−^ mice was a result of enhanced tolerance is not known. If so, R-Ras might oppose Rap1 by competing for common downstream effectors. Further characterization of IL-2 gene expression in T cells in mice from the GVHD model is necessary.

We previously reported that R-Ras is required for CD4^**+**^-mediated maturation of DCs by promoting the formation of immune synapses [18]. Our results indicated that *Rras* ^−/−^ T cells have normal proliferative capacity when stimulated with several soluble immune regulators. Intriguingly, T cells that lack R-Ras have proliferative defects *in vivo* or when co-mixed with APCs. To explain this discrepancy, we speculate that R-Ras expression in T cells may be required for immune synapse formation with APCs and that this expression is essential for T cell proliferation. A previous study reported on the role of lymphotoxin-β (Lt) secreted by DCs in promoting HEV maturation [23]. Indeed, similar to *Rras* ^−/−^ mice, mice with Lt specifically knockout in DCs have reduced cellularity of PLNs. Although we observed reduced DC numbers in *Rras* ^−/−^ mice, it is unclear whether this is responsible for the smaller lymph node phenotype. Another possibility is that R-Ras may directly regulate Lt production in DCs. Further studies are needed to test these hypotheses.

HEVs are composed of specialized endothelial cells in the T cell zones of PLNs [21]. Members of the TNF family of lymphokines, such as TNFα and Lts, promote the differentiation of stromal and endothelial components of PLNs [32]. These factors also up-regulate adhesion molecules, such as ICAM-1 and LFA-1, as well as glycans, on endothelial cells to capture homing T cells. R-Ras is highly expressed in endothelial cells and vascular smooth muscle cells [17]. It also plays a homeostatic role in the vasculature by conferring junctional integrity to endothelial cells [19]. The immature HEV observed in R-Ras-null mice may, therefore, be a direct effect of R-Ras knockout in endothelial cells. It will eventually be necessary to define the role of R-Ras in different cell types (T cells, endothelial cells, DCs) by cell type-specific knockout of R-Ras.

As a small G-protein, the upstream regulator of R-Ras is elusive. The common consensus is that R-Ras, unlike classical Ras, is not regulated by growth factors. Instead, signaling molecules involved in cell-cell adhesion have been implicated; these include semaphorins [15], EphB2 [33], and Notch1 [16]. Our data provide the first demonstration of the chemokine, CCL21, in stimulating the GTP-loading of R-Ras. Similar to Rap1A, the kinetics of R-Ras activation by this chemokine are rapid but transient, and the activation returns to the background level within 5 min. The low fold-increase in R-Ras-GTP may reflect the poor sensitivity of the assay system in the measurement of endogenous proteins. Alternatively, the fraction of R-Ras activated in T cells is very small. This modest activation of R-Ras was also observed in the LPS-stimulated DCs that we previously reported [18].

The reduced binding of *Rras* ^−/−^ T cells to ICAM-1 may be explained by two plausible mechanisms. First, the surface expression levels of the CD11a subunit of LFA-1 were decreased in *Rras* ^−/−^ T cells. Second, R-Ras may serve as a signaling intermediate between chemokine receptors and integrin activation. The averaged reduction of 8.8% in CD11a expression in *Rras* ^−/−^ T cells may not account for the 14.5% attenuation in the adhesion to ICAM-1. Therefore, the reduced ICAM-1 binding observed with R-Ras-null T cells most likely due to a combination of reduced LFA-1 expression and activation.

If R-Ras mediated LFA-1 activation via an inside-out-signaling mechanism, it was not likely due to alterations in Akt or Erk1/2 activation because their activation states were unaffected in R-Ras-null T cells after CCL21 stimulation. The inside-out signaling pathway that links the chemokine receptor to integrin activation involves the activation of protein kinase C leading to Rap activation [34]. Rap binds to RapL and in turn enhances LFA-1 membrane recruitment and avidity [30]. The binding of the adaptor protein Kindlin3 to the β2 subunit of LFA-1 displaces the cytoskeleton protein filamin from restraining integrin activation [35]. Interestingly, R-Ras has been shown to bind filamin [36]. Whether R-Ras can directly regulate these signaling events in T cells will be a subject for future investigation. In addition, whether reduced ICAM-1 binding of *Rras* ^−/−^ T cells can affect rolling and firm adhesion to HEV remains to be examined. We demonstrated in a mouse endothelial cell line that *Rras* ^−/−^ T cells have reduced adhesion. However, it is uncertain whether this reduction is mediated by the interaction between LFA-1 and ICAM-1. Reduced surface expression of CD62L in *Rras* ^−/−^ T cells may also play a role. Of note, the PI3-Kδ isoform has been known to regulate CD62L shedding and transcription [37]. For instance, up-regulation of ADAM17, a receptor-type metalloprotease, may enhance CD62L shedding in *Rras* ^−/−^ T cells [38]. Because, R-Ras has been known to bind and stimulate PI3-Kδ isoform [39], perturbation of the PI3-Kδ activation state in R-Ras-null T cells may alter the CD62L levels.

Taken together, these data provide evidence for a role of R-Ras in the mediation of chemokine receptor-mediated signaling in migrating T cells in the HEV. Our results reinforce the general concept that diverse small GTPases of the Ras subfamily play distinct roles in the propagation of physiologic signals.

## Supporting Information

S1 Fig Immune cell development and functions of *Rras* ^−/−^ mice. Indicated lymphoid organs from *Rras* ^**+/+**^ (WT) and *Rras* ^−/−^ (KO) mice were analyzed with flow cytometry for subpopulations of T cells. Results are presented as percentage (%) of total from three to six mice per group. *Bars*, SE.
(TIF)

S2 Fig Normal body mass of *Rras* ^−/−^ mice. Male and female *Rras* ^**+/+**^ (WT) and *Rras* ^−/−^ (KO) mice were weighted at 6 and 9 weeks of age. The number of animals used per group is indicated. *Bars*, median values.
(TIF)

S3 Fig Reduced CD62L populations in *Rras* ^−/−^ mice. The fraction of CD62L^**+**^ in CD4^**+**^ and CD8^**+**^ T cells from spleen and lymph nodes (LN) of *Rras* ^**+/+**^ and *Rras* ^−/−^ mice was analyzed with flow cytometry. Results from 12 mice per group were quantified and depicted. *Bars*, SD, two-tailed *t*-test. **p<0.005, ***p<0.0001.
(TIF)
