# Supplementary figures and images for: R-Ras Regulates Murine T Cell Migration and Intercellular Adhesion Molecule-1 Binding
Source: PLoS One. 2015 Dec 28;10(12):e0145218. doi: 10.1371/journal.pone.0145218 (PMC4692399; doi:10.1371/journal.pone.0145218)

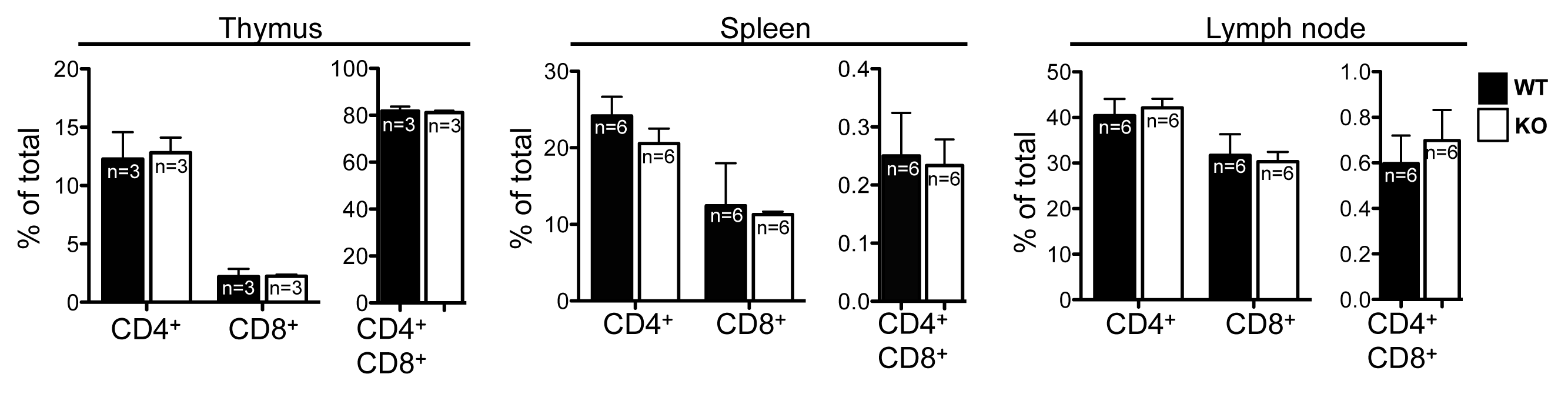

Supplement: S1 Fig — Indicated lymphoid organs from Rras +/+ (WT) and Rras −/− (KO) mice were analyzed with flow cytometry for subpopulations of T cells. Results are presented as percentage (%) of total from three to six mice per group. Bars, SE. (TIF) [file pone.0145218.s001.tif]

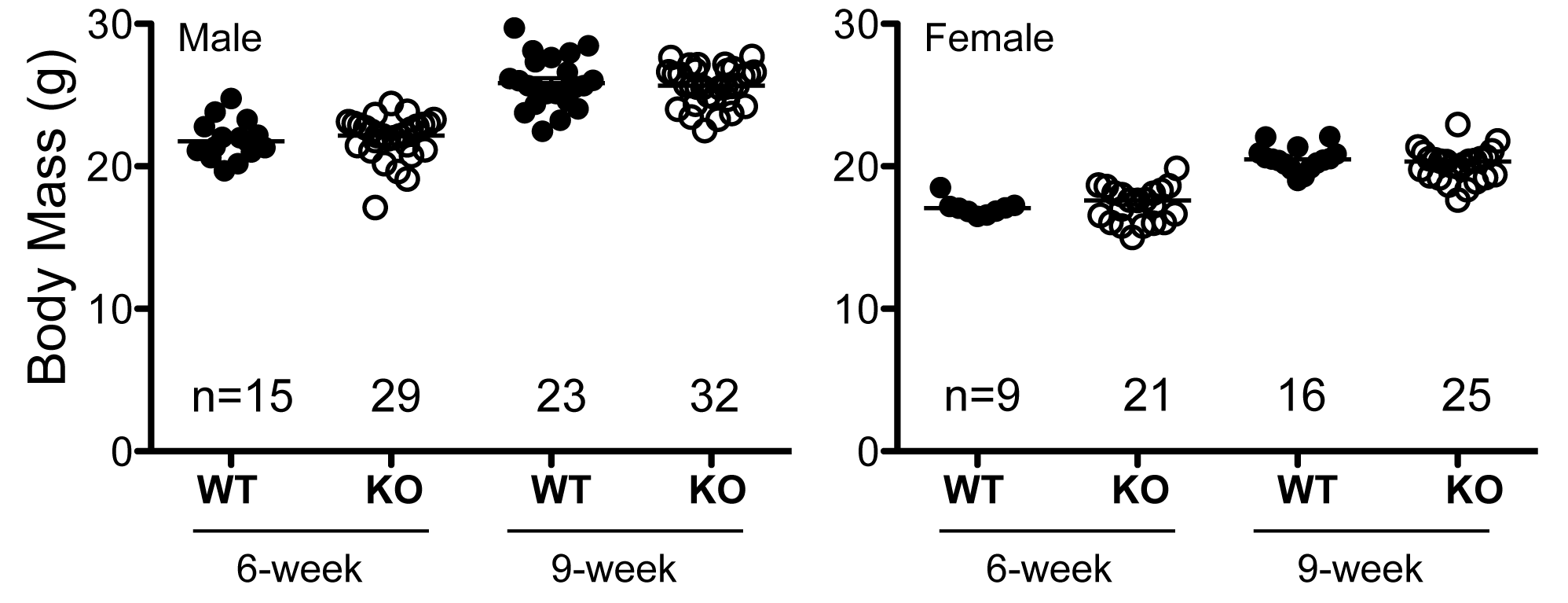

Supplement: S2 Fig — Male and female Rras +/+ (WT) and Rras −/− (KO) mice were weighted at 6 and 9 weeks of age. The number of animals used per group is indicated. Bars, median values. (TIF) [file pone.0145218.s002.tif]

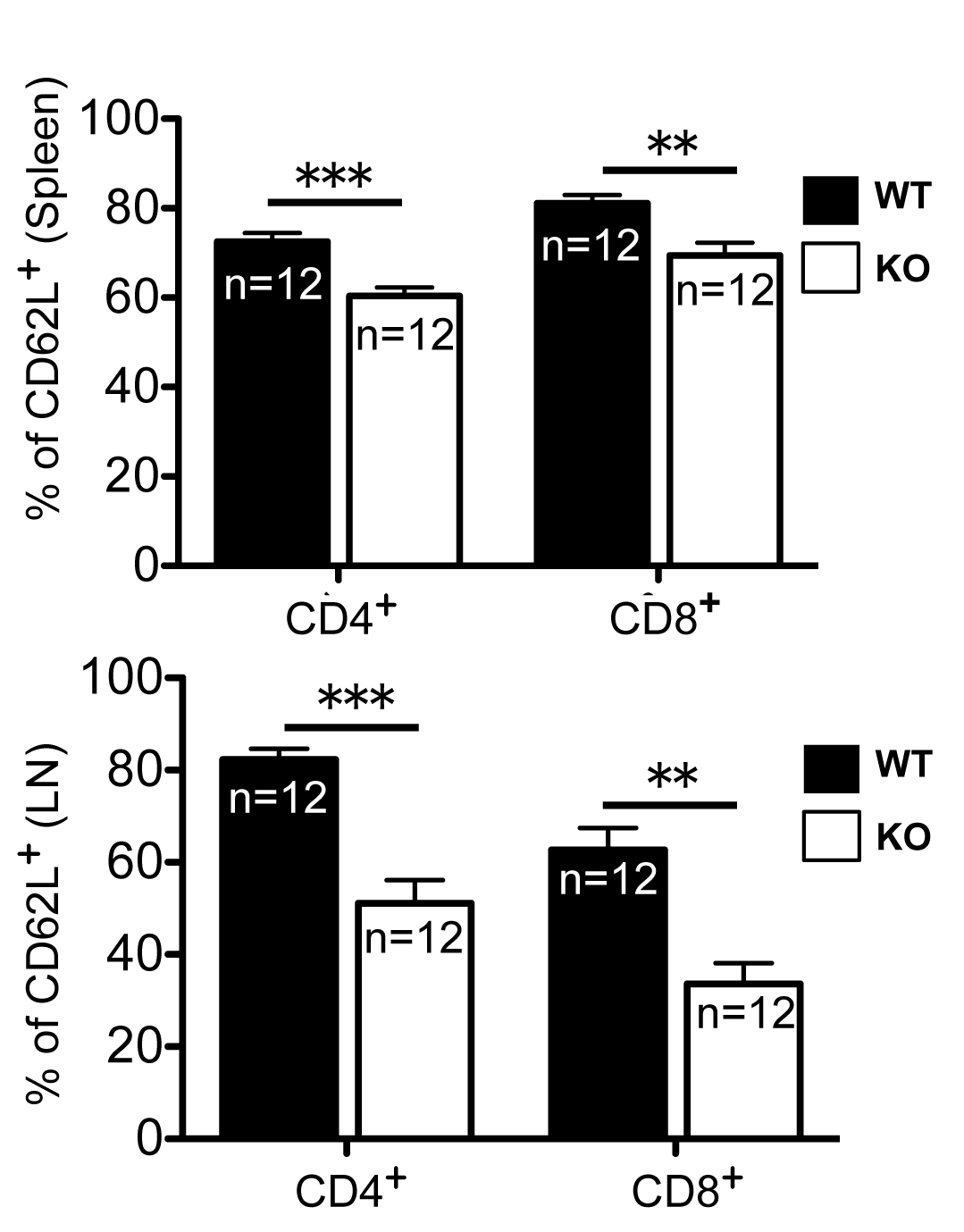

Supplement: S3 Fig — The fraction of CD62L+ in CD4+ and CD8+ T cells from spleen and lymph nodes (LN) of Rras +/+ and Rras −/− mice was analyzed with flow cytometry. Results from 12 mice per group were quantified and depicted. Bars, SD, two-tailed t-test. **p<0.005, ***p<0.0001. (TIF) [file pone.0145218.s003.tif]
